# HPLC for at-line reaction monitoring and purification improves yield and purity of tRNA

**DOI:** 10.3389/fmolb.2024.1443917

**Published:** 2024-09-27

**Authors:** Polona Megušar, Ewen D. D. Calder, Tina Vodopivec Seravalli, Sergeja Lebar, Louise J. Walport, Rok Sekirnik

**Affiliations:** ^1^ Sartorius BIA Separations d.o.o., Ajdovščina, Slovenia; ^2^ Department of Chemistry, Molecular Sciences Research Hub, Imperial College London, London, United Kingdom; ^3^ Protein-Protein Interaction Laboratory, The Francis Crick Institute, London, United Kingdom

**Keywords:** tRNA, *in vitro* transcription, HPLC, chromatography, anion exchange

## Abstract

Engineered transfer RNA is an emerging therapeutic modality, particularly suited to treatment of diseases caused by genetic disorders based on premature termination codons, frameshifts, or missense mutations. It is also extensively used in reprogramming of *in vitro* translation systems to generate non-canonical amino acid-containing proteins and peptides, such as in mRNA display. Due to its length, chemical synthesis of tRNA is challenging and production of engineered tRNA at scale is currently limited to *in vitro* transcription from a DNA template. Previously, the highest reported *in vitro* transcription yield was 2.5 g/L, significantly below the industry standard for mRNA production of 7–10 g/L. To improve this process, we implemented monitoring of nucleoside triphosphate consumption and tRNA production during *in vitro* transcription, using at-line high-performance liquid chromatography, with a monolithic solid phase. This allowed for optimization of nucleoside triphosphate concentration, reduction of the *in vitro* transcription time to <4 h, and improvement of yield up to 4.7 g/L. A step-elution purification on a DEAE chromatographic monolith with >90% step yield was then developed. These improvements in the production and purification of tRNA represent an important step in facilitating production of tRNA for research purposes, and provide a method for purification of therapeutic tRNAs that is scalable and compatible with Good Manufacturing Practice requirements for clinical production.

## Introduction

Transfer RNA (tRNA) is a key component of the protein synthesis machinery, acting as the adaptor molecule that decodes mRNA into amino acid sequences. Recent advances have unveiled further tRNA roles beyond their canonical function in translation, including regulation of gene expression, involvement in disease pathogenesis, and their potential as therapeutic agents (Anastassiadis and Köhrer, 2023; Berg and Brandl, 2021; Coller and Ignatova, 2023).

The therapeutic potential of tRNAs is significant, as they can be engineered to correct genetic mutations that cause disease. Mutations within protein-coding sequences can lead to various pathologies by creating premature termination codons, frameshifts, or missense mutations, all of which can be targeted by specifically designed tRNAs. For instance, engineered tRNAs can facilitate read-through of premature termination codons, adjust reading frames disrupted by frameshift mutations, or correct missense mutations, thereby restoring the synthesis of functional proteins (Anastassiadis and Köhrer, 2023; Coller and Ignatova, 2023).

tRNA therapeutics offer an approach to treating genetic diseases based on targeting the mutation itself rather than the affected gene. This strategy could transform the treatment landscape for patients with rare and ultrarare diseases, which often lack viable therapeutic options due to the small patient populations and the high specificity of gene-targeted treatments. Engineered tRNAs have been shown to suppress premature termination codon mutations effectively, both *in vitro* and *in vivo* (Albers et al., 2023). Moreover, the ability to deliver tRNAs directly as RNA (formulated as lipid nanoparticle or exosome) or via viral vector (e.g., AAV) expands the potential delivery options and facilitates the development of versatile therapeutic platforms (Coller and Ignatova, 2023).

Synthetic tRNA are also used extensively for genetic code reprogramming in chemical biology and drug discovery applications. A range of methods have been developed to couple non-natural amino acids to tRNA, including by chemoenzymatic synthesis, use of engineered aminoacyl tRNA synthetases or use of evolved ribozymes, known as flexizymes (Forster et al., 2003; Heckler et al., 1984; Murakami et al., 2006; Sigal et al., 2024). Introduction of these artificially aminoacylated tRNA into *in vitro* translation reactions allows synthesis of peptides and proteins containing a vast variety of non-natural amino acids that can augment their functions (Zhou and Obexer, 2024). This has been exploited extensively, both in industry and academia, through combination with mRNA display for the discovery of *de novo* cyclic peptides (Huang et al., 2019; Josephson et al., 2005; Ohta et al., 2023; Yamagishi et al., 2011).

Due to its size (70–90 nucleotides), tRNA is too long for large scale *de novo* production by chemical synthesis. Instead, it is primarily produced by an *in vitro* transcription (IVT) reaction, an RNA polymerase-catalyzed incorporation of nucleoside triphosphates (NTPs) into a nascent RNA chain guided by a DNA template (Milligan et al., 1987). Alternatively, tRNA can also be produced by *in vivo* overproduction (Perona et al., 1988). Optimizing IVT conditions for high yield, as well as developing efficient purification methods is critical for cost-effective production of mRNA, self-amplifying RNA (saRNA) and circular RNA (circRNA) therapeutics (Rosa et al., 2021). For tRNA, only few reports have addressed optimization of IVT reaction, of which the key study described optimization of *Escherichia coli* tRNATrp production by IVT to 2.5 g/L by fine-tuning the concentrations of T7 RNA polymerase, DNA template, NTPs, and magnesium, using a combination of incomplete factorial design and response surface methodology (Yin and Carter, 1996). The study used densitometry as well as a radioactive labelling approach to quantitate yield, both of which could lead to potential quantification errors. Other analytical methods have since been applied to monitor the yield of IVT reactions in real time (reviewed in Lee et al., 2023): light-up RNA aptamer coupled with fluorescent dye pairs (Höfer et al., 2013; Kartje et al., 2021; Valentini et al., 2022) and fluorophore-labeled antisense probe-based methods (Dunkak et al., 1996). High-performance liquid chromatography (HPLC)-based methods able to monitor NTP consumption as well as RNA production in near-real time have recently been developed (Pregeljc et al., 2023; Welbourne et al., 2024) with resolution-times of 3–6 min and total run-times of ∼8 min, thereby enabling IVT reaction optimization based on kinetics of NTP consumption. To our knowledge, none of these methods have been applied for monitoring of IVT reactions for production of tRNA.

For *in vitro* or *in vivo* use, tRNA needs to be purified from IVT reaction mixture to remove residual NTPs, enzymes and DNA template. The most common approach to purify synthetic tRNA is by polyacrylamide gel electrophoresis and excision of the tRNA band from the gel (Avcilar-Kucukgoze et al., 2020). This method is highly selective for target tRNA, potentially allowing single-nucleotide resolution. However, PAGE gels are poorly scalable beyond laboratory scale, and require handling toxic acrylamide during preparation of gels.

A widely used scalable purification approach, which can be employed at clinical and commercial manufacturing scales under Good Manufacturing Practice compliance, is chromatography. Various chromatographic techniques have been used to purify tRNA, either IVT-derived (defined tRNA sequence) or from *E. coli* extract, including ion exchange (Guenther et al., 1988), reverse-phase HPLC (RP-HPLC) (Cayama et al., 2000) and hydrophobic interaction chromatography (HIC) (Mesters et al., 1994). More selective approaches including use of dihydroxyboryl cellulose (McCutchan et al., 1975), which bind the cis-diol group of the 3′-terminal ribose in unaminoacylated, but not aminoacylated tRNA, have also been explored, however, these are not used in practice as dihydroxyboryl cellulose is not a commercially available chromatography media. Seminal preparative scale tRNA purification from IVT was reported with weak anion exchanger (DEAE Sepharose), which suffered from broad elution peaks and was not able to fully separate DNA template from tRNA (Easton et al., 2010). Chromatographic resolution was significantly increased with a strong anion exchanger (MonoQ) enabling baseline separation of tRNA from DNA template (Koubek et al., 2013), and although quantification of tRNA in IVT mixtures was shown, NTPs could not be quantified and the separation was too slow to be useful for at-line IVT monitoring. MonoQ was reported to require multiple elutions to achieve full RNA recovery, suggesting that strong AEX could present issues for tRNA recovery (Pikovskaya et al., 2009). We reasoned that resolution and recovery of tRNA could be improved, and purification time decreased, with use of chromatographic monoliths, stationary phases consisting of a single piece of highly porous polymer with open, ligand-functionalized pores forming interconnected channels which provide binding surface for analytes (Podgornik et al., 2013). Due to their architecture, monoliths exhibit high binding capacity for large biomolecules (plasmid DNA, mRNA, virus particles) and mass transport based on convection, which results in flow-rate independent chromatographic resolution and binding capacity. Analytical anion exchange monolith can resolve a mixture of oligonucleotides with single-nucleotide resolution in 10 min (Yamamoto et al., 2009).

We recently reported how the high resolution at short run times of monoliths can be harnessed to analytically resolve NTPs from mRNA to monitor IVT reaction for production of mRNA (Pregeljc et al., 2023). In the present study we expand the scope of the analytical strategy to increase yield of IVT-produced tRNA by analyzing the consumption of NTPs and optimizing reagent concentrations and reaction times. Further, we verify that recently developed monolith chromatographic supports which showed high recovery of mRNA and saRNA at room temperature and neutral pH (Megušar et al., 2023; Miklavčič et al., 2023) can also be used to purify tRNA, and compare their purification efficiency to more traditional anion exchange ligands (e.g., diethylaminoethyl DEAE) with high (single-nucleotide) resolution for oligonucleotides at short run times (Podgornik et al., 1999; Yamamoto et al., 2007). We demonstrate that applying convective monolith stationary phases, with flow-rate independent binding capacity and resolution, could shorten tRNA purification without sacrificing tRNA purity with the potential for industrial applications in the future.

## Materials and methods

### At-line monitoring of *in vitro* transcription by HPLC

40 U/µL RNase inhibitor, 100 U/µL pyrophosphatase, 50 U/µL T7 RNA polymerase, ATP, UTP, CTP and GTP (100 mM stocks) were purchased from Jena Biosciences or Mebep Bioscience, China. 1 M MgCl_2_ was purchased from Invitrogen, USA, 10 × IVT buffer (400 mM Tris, 20 mM spermidine, 10 mM DTT, pH 7.9) was prepared in-house. All IVT reagents listed except enzymes were preheated to 37°C, mixed in a 1.5 mL plastic tube in Thermomixer™C (Eppendorf, Germany) and, after addition of enzymes, incubated at 37°C with shaking at 300 rpm unless otherwise stated. For sampling, 2 µL aliquots were quenched with 2 µL of 100 mM EDTA pH 8.0 for HPLC analytics at defined timepoints. tRNA production and NTP consumption were monitored by CIMac PrimaS™ (Sartorius BIA Separations, Slovenia) every 30–60 min.

IVT reaction optimization for tRNA was based on reference protocol described in Table 1.

**TABLE 1:** Reference IVT reaction conditions for tRNA production. GMP was not used in the reaction.

Reagent	Final concentration
Nuclease free water	
10x IVT buffer	1x
RNAse inhibitor 40 U/µL	1 U/µL
MgCl_2_	22.5 mM
NTP (each)	3.75 mM
DNA template	4.3 ng/μL
Pyrophosphatase, 100 U/mL	1 U/mL
T7 RNA polymerase 50 U/µL	10 U/µL

### CIMac PrimaS analysis for determination of tRNA concentration and NTP consumption

HPLC analysis for mRNA quantification and determination of NTP consumption was performed as previously reported (Skok et al., 2022). PATfix® 2.0 software (Sartorius BIA Separations, Slovenia) was used for instrument control, data acquisition and data analysis. A purified tRNA standard was used for calibration of the UV signal corresponding to tRNA. Linearity of signal responses (tRNA, UTP/CTP, GTP, ATP) is shown in Supplementary Figure S1; samples for HPLC analysis were diluted accordingly.

### Purification development

In a typical chromatographic experiment, a freshly prepared IVT reaction mixture was diluted at least 10-fold with selected mobile phase A (MPA) i) 50 mM citric acid pH 5 for Swiper and ii) 100 mM Tris, 300 mM guanidine hydrochloride (GuHCl), pH 8 for DEAE) to provide pH buffering and binding conditions. Chromatographic purification was performed using PATfix system (Sartorius BIA Separations, Slovenia) equipped with quaternary pump and a multiwavelength UV-Vis detector (10 mm flow cell path length). PATfix 2.0 software (Sartorius BIA Separations) was used for instrument control and data acquisition. Column was equilibrated with at least 10 CV selected mobile phase B (MPB) [i) 50 mM citric acid, 0.3 M NaCl pH 5 for Swiper and ii) 100 mM Tris, 300 mM GuHCl, 700 mM NaCl, pH 8 for DEAE] followed by at least 10 CV of selected MPA. Sample, usually with tRNA concentration between 300 and 400 μg/mL, was loaded onto CIM discs or CIMmultus® Swiper or DEAE monolith column (0.1 or 1 mL, respectively) with 2 μm channel size (Sartorius BIA Separations, Ajdovščina, Slovenia) at 1–10 column volume per min (CV/min) and UV absorbance was monitored at 260 nm. After UV260, conductivity and pH signal stabilized, where indicated, wash and/or elution steps where performed: a) CIM Swiper: high salt wash (MPB), followed by elution step with 100 mM sodium phosphate pH 7.5 and cleaning in place step (CIP) for column sanitization with 0.1 M NaOH and 1 M NaCl; b) DEAE: elution with linear gradient from MPA to MPB in 15 min or elution in step gradient described below in spin column section.

### tRNA purification with spin columns

Samples containing tRNA were diluted 10-fold in 100% MPA. Sample was then transferred onto a prototype CIM DEAE spin column (0.1 mL bed volume) and loaded by centrifuging at 1,200 rpm for 3 min. Column was then washed with 10 CV of MPA (3,000 rpm for 2 min) to remove NTPs and tRNA fragments. Permeate was collected in the collection tube and transferred to a separate tube for analytical purposes. Next, 5 CV of elution buffer 1 (70% MPA, 30% MPB) was used to wash the column. Elution was transferred to a separate tube. 5 CV of elution buffer 2 (60% MPA, 40% MPB) was used to elute the tRNA. The elution, which contained final purified tRNA, was transferred into a new tube. The column was stripped with 5 CV of 100% MPB. Spin columns were sanitised with 5 CV of 0.1 M NaOH, 1 M NaCl and regenerated with 10 CV of MPA before next use.

### Aminoacylation of tRNA

Flexizyme-mediated aminoacylation reactions were performed as described previously (Goto et al., 2011a; Goto et al., 2011b). Aminoacylation was performed by mixing 5 mM amino acid cyanomethyl ester (Biotin-l-Phe-CME or d-Phe-CME) with 600 mM MgCl_2_, 20% DMSO, 25 μM eFx and either 25 μM initiator tRNA^fMet^_CAU_, 25 μM elongator tRNA^AsnE2^_CAU_ or 25 μM elongator tRNA^AsnE3^_CAU_ in 50 mM HEPES-KOH (pH 7.5). The mixture was incubated for 2 h on ice, precipitated with sodium acetate and ethanol and resuspended in 1 mM NaOAc. Solution at 250 μM is assumed to be 50% loaded with amino acid and is used without further purification.

### *In vitro* translation of an mRNA template for MALDI-TOF MS

mRNA templates used for *in vitro* translation experiments encoded for either MGSVSGWRLFKKISGSGSGS for initiator testing or MGSMGSVSGWRLFKKISGSGSGS for elongator testing.

Translations were performed using solution B from a PURExpress (NEB), (Δaa, ΔtRNA) kit and solA (composition described in Supplementary Material). Working on ice, the mRNA template (5 μM, 1.0 μL), 19 amino acid mix (ΔMet, 5 μM each amino acid, 0.50 μL), aminoacylated tRNA (125 μM, 1.0 μL), water (0.22 μL), solA (0.78 μL) and PURExpress (Δaa, ΔtRNA) solution B (1.5 μL) were combined. The solution was mixed by pipette and incubated with shaking at 37 °C for 1 h.

## Results

### IVT optimization

We aimed to develop an optimized protocol for the synthesis and purification of tRNA by IVT. Initial efforts focused on IVT optimization following an HPLC-based at-line IVT monitoring approach previously reported for mRNA. The methodology employed a rapid HPLC analytical method based on multimodal anion exchange/hydrogen bonding ligand PrimaS which separates NTPs from DNA and RNA. This method has been employed to increase IVT yield for mRNA and saRNA (Pregeljc et al., 2023; Skok et al., 2022), but has not been applied for monitoring IVT to produce shorter RNA constructs such as tRNA.

The starting point for optimization was a standard IVT protocol for tRNA production commonly used to produce tRNA (Goto et al., 2011a; Milligan and Uhlenbeck, 1989). The historical tRNA IVT protocol employs guanosine monophosphate (GMP) to incorporate a 5′-monophosphate (instead of a 5′-triphosphate incorporated by guanosine triphosphate (Milligan and Uhlenbeck, 1989; Sampson and Uhlenbeck, 1998)). Since multiple studies demonstrated that a 5′-phosphate does not have a significant impact on efficiency of tRNA aminoacylation (Fechter et al., 1998; Sampson and Uhlenbeck, 1998) or *in vitro* translation (Sprinzl and Graeser, 1980), we performed IVT optimization without GMP, using NTPs only, translating an initiator tRNA template (tRNA^fMet^_CAU_, INI).

**FIGURE 1:**
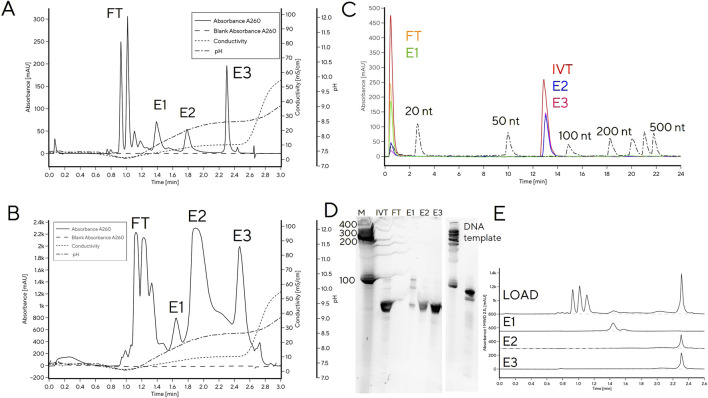
Separation of IVT mixture containing using a tRNA CIMac PrimaS analytical HPLC column. **(A)** Analytical loading (1 μg) onto CIMac PrimaS; **(B)** 50 μg loading onto CIMac PrimaS; **(C)** overlay of IP-RP analytical chromatograms of CIMac PrimaS elution fractions (FT, E1-E3) and molecular weight standards, **(D)** 10% TBE-Urea PAGE gel of CIMac PrimaS elution fractions, **(E)** CIMac PrimaS analytical chromatograms of CIMac PrimaS elution fractions. M: molecular weight marker (Small RNA ladder, Agilent), IVT: quenched IVT mixture, FT: column flow-through fraction, L: load, E1-E3 elution fractions E1-E3 from 50 μg loading onto CIMac PrimaS.

A reference IVT reaction mixture after 3 h reaction time was separated into multiple peaks by CIMac PrimaS. Retention times (RT) corresponding to NTPs were easily identifiable, while the remaining peaks presumably corresponded to DNA and tRNA (1.4 min (E1), 1.8 min (E2) and 2.3 min (E3), Figure 1). Only the RT of E3 coincided with typical RT of mRNA. To confirm the identity of chromatographic peaks, tRNA loading on the column was increased to 50 μg to allow collection of elution fractions for analysis by RP-HPLC, PAGE gel and CIMac PrimaS (Figures 1B–E). Due to higher mass loading onto the analytical column, the resolution between peaks decreased (Figure 1B) compared to analytical loading (Figure 1A) but the order of elution was identical.

**FIGURE 2:**
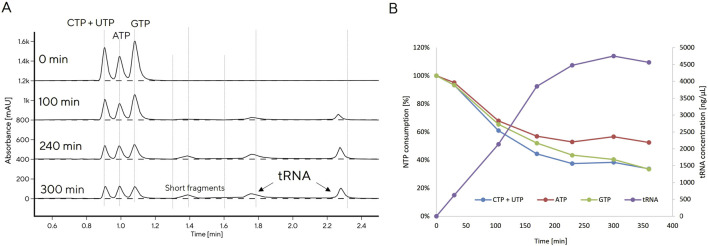
CIMac PrimaS monitoring of tRNA production with *in vitro* transcription (IVT 6) reaction **(A)**, tRNA production and NTPs consumption monitored at-line with CIMac PrimaS **(B)**.

The two major elution peaks (E2 and E3) were indistinguishable by IP-RP and PAGE (Figures 1C, D), and corresponded to tRNA of target size (76 nt, determined by IP-RP). Analytical profiles of elution fractions E2 and E3 were consistent with tRNA of same length, therefore the combined integral of peak areas was used for quantification of tRNA in IVT mixtures when IVT reactions were monitored at-line for tRNA content and NTP concentrations (Figure 2) and in calibration curves (Supplementary Figure S1). E1 did not bind to IP-RP column under analytical binding conditions (Figure 1C; RNA chain length >20 nt is required for binding); PAGE band of E1 was consistent with migration of DNA template (Figure 1D); and the CIMac PrimaS elution profile was consistent with elution of DNA template or short RNA fragments (Figure 1E). We concluded that E1 corresponds to DNA template and/or short RNA fragments, but not target tRNA.

The reference IVT protocol resulted in production of 1.5 g/L tRNA after 3 h. GTP was completely consumed, indicating that GTP was limiting for tRNA production (data not shown). We increased GTP concentration in the IVT reaction, initially in batch, then in fed-batch mode (Table 2). DNA concentration was also varied based on reports that it may impact IVT productivity (Yin and Carter, 1996), as were the concentrations of other NTPs (Table 2).

**TABLE 2:** Optimization of IVT parameters for production of tRNA.

Entry	Batch (B)/Fed-batch (FB)	ATP (mM)	GTP (mM)	GMP (mM)	CTP and UTP (mM, each)	MgCl_2_ (mM)	DNA (ng/μL)	T7 (U/mL)	tRNA yield after 4 h (g/L)	tRNA yield overnight (g/L)
Reference (IVT 1)	B	3.75	3.75	—	3.75	22.50	4.3	10	1.5	—
IVT 2	B	6.00	6.00	—	6.00	25.00	4.0	10	1.8	—
IVT 3	B	4.00	5.50	—	4.00	22.50	8.0	10	2.9	—
IVT 4	B	6.00	12.00	—	6.00	27.00	3.0	10	4.1	4.5
IVT 5	B	4.50	12.00	—	5.00	23.90	3.0	10	4.0	4.2
IVT 6	B	6.00	12.00	—	6.00	27.00	5.0	12	4.7	4.8
IVT 7	FB	8.00	8.00^b^	—	8.00	30.00	5.0	10	3.2	4.7
IVT 8	FB	6.00^a^	10.00^b^	—	6.00^c^	30.00	5.6	10	3.5	4.4
IVT 9	B	6.00	6.00	5.00	6.00	25.00	4.0	10	1.5	—

^a^ Feed additions of ATPs are marked with a.

^b^ Feed additions of GTP are marked with b.

^c^ Feed additions of CTP and UTP are marked with c.

An increase in GTP from 3.75 to 6 mM led to a moderate increase in yield from 1.5 to 1.8 g/L (IVT 2). Yield could also be doubled if DNA template concentration was doubled (IVT 3), though this effect is likely limited to low overall NTP concentrations. A further increase in NTP to 6 mM and GTP concentration to 12 mM (IVT 4) yielded 4.1 g/L tRNA, surpassing the highest previously reported yield of 2.5 g/L (Yin and Carter, 1996). Comparable yield (4.0 g/L) could be achieved if GTP was maintained at 12 mM while other NTPs were reduced to 4.5–5.0 mM (IVT 5). When more DNA template (5 ng/μL) and more T7 polymerase (12 U/mL) was used (IVT 6), the reaction proceeded faster and increased the yield even further: 4.7 g/L was reached after 4 h and did not increase further with overnight incubation. This result was not surpassed by fed-batch approach (IVT 7, IVT 8) which resulted in a decreased reaction rate, but not overall yield, possibly due to additional dilution of reaction components which slow the reaction; tRNA was produced more slowly after feeding but the reaction could still reach a high yield if incubated overnight. Nearly 50% higher yield was observed if fed-batch reactions were incubated overnight, whereas only a 10% yield increase was observed when leaving batch reactions overnight, which already reached >4 g/L in 3 h.

We tested our optimized IVT protocol on two variant elongator tRNAs (tRNA^AsnE2^_CAU_, ELO2, and tRNA^AsnE3^_CAU_, ELO3, Supplementary Table S1). These variant tRNAs bind to elongation factor thermo unstable (EF-Tu) during translation with different affinities and allow incorporation of some unnatural amino acids to occur with higher yields (Iwane et al., 2021). IVT protocol optimized on INI resulted in a more modest increase in yield to 2.5 g/L for two other tRNA constructs (ELO2, ELO3) with different AUCG content (Supplementary Table S1), while reference protocol resulted in a comparable yield to INI (1.8 g/L; Supplementary Figure S2). It is notable that ELO2 and ELO3 share high sequence homology to each other, but differ in AUCG content from INI.

In IVT reactions for ELO2/3, CIMac PrimaS detected an increase in area of chromatographic peak at 1.4 min (Supplementary Figure S3A), which increased with a higher rate compared to tRNA peak (2.3 min; Supplementary Figure S3B). We increased sample loading to CIMac PrimaS to isolate fractions (Supplementary Figure S3C) and confirmed that the peak corresponds to RNA fragment of 20–40 nt (Supplementary Figure S3D–F), suggesting that ELO2/ELO3 constructs under ‘high yield IVT conditions’ led to a higher amount of aborted transcripts. We can conclude that IVT conditions to optimize tRNA yield may be construct-specific, at least for sequences with significantly different AUCG content, underscoring the importance of IVT monitoring when optimizing reactions to distinguish between full-length and abortive transcripts.

**FIGURE 3:**
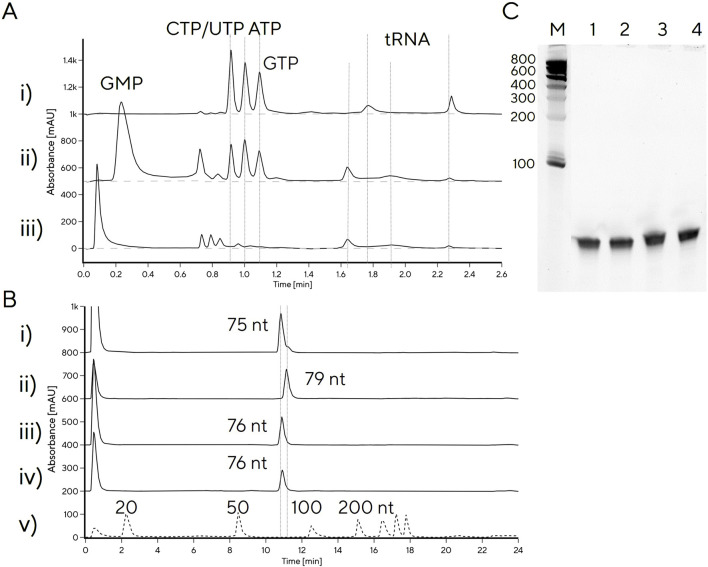
Analytical profiling of tRNA with variable 5′-phosphorylation status. **(A)** CIMac PrimaS HPLC analytical profiles of **(i)** tRNA produced with NTPs only; ii) tRNA produced with GMP, iii) tRNA produced with NTPs and treated with 5′-phosphatase. **(B)** CIMac SDVB RP-HPLC profiles of **(i)** IVT with GMP, ii) IVT without GMP, iii) IVT without GMP, treated with 5′-phosphatase (without purification); iv) tRNA produced without GMP, purified by Swiper and treated with 5′-phosphatase. **(C)** PAGE gel of 1) IVT treated with phosphatase, 2) Swiper-purified tRNA treated with phosphatase, 3) IVT without GMP, 4) IVT with GMP.

As most tRNA IVT protocols still include GMP (Goto et al., 2011a; Korenčić et al., 2002; Milligan and Uhlenbeck, 1989), we also investigated what effect addition of GMP would have on reaction yield. Adding GMP to reaction conditions of IVT 9 yielded 1.5 g/L tRNA compared to 1.8 g/L with NTPs only, likely due to competition with GTP for initiation of transcription. We noticed that the tRNA produced with GMP (IVT 9) exhibited a different chromatographic profile on CIMac PrimaS compared to tRNA produced without GMP, suggesting that retention times of the tRNA peaks depend on 5′-phosphorylation status. To verify this, tRNA was treated with 5′-phosphatase, which converts a 5′-triphosphate to a 5′-monophosphate. tRNA produced with GMP or treated with phosphatase exhibited a broader peak between 1.9 min, instead of the characteristic tRNA peak at 2.2 min (Figure 3A). It was surprising to observe that a difference in 5′-phosphorylation leads to observable differences in elution profile and suggests that 5′-phosphorylation state plays a significant role in binding to the stationary phase. This could potentially be exploited further for determination of 5′-phosphorylation status of tRNA, in combination with IP-RP, which also distinguished between 5′-mono- and triphosphate tRNA. In IP-RP analytical chromatograms, the 5′-monophosphate tRNA eluted earlier than 5′-triphosphate tRNA (Figure 3B), the GMP-tRNA peak contained a shoulder at a higher retention time, indicating incomplete 5′-monophosphate incorporation. Denaturing PAGE electrophoretic mobility of all three tRNAs were indistinguishable (Figure 3C), consistent with unchanged tRNA length.

### tRNA purification

**FIGURE 4:**
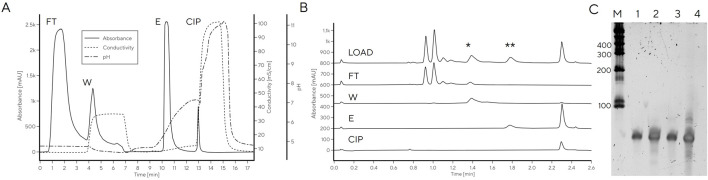
**(A)** Purification of tRNA from IVT with CIMmultus Swiper. Purification was performed as described in the material and methods **(B)** CIMac PrimaS analytics of fractions collected from tRNA purification on CIMmultus Swiper. **(C)** 8% Urea PAGE. FT: Flow-through. W: Wash. E: Elution. CIP: cleaning in place. M: Low range RNA ruler. 1–2: Gel-purified tRNA (50 and 250 pmol, respectively). 3–4: Swiper-purified tRNA (50 and 250 pmol, respectively).

We next investigated whether HPLC separation observed with analytical PrimaS method could be extended to a chromatographic purification method to avoid the slow and inefficient gel purifications that are commonly used for tRNA purification. A larger mass of tRNA could not be applied to PrimaS operated with a pyrophosphate gradient, as used for analytical separation, without losing resolution between peaks (e.g., Figure 1B). Use of a non-pyrophosphate buffer system was also unfeasible as pH > 10.5 would be required for RNA elution from PrimaS as previously reported (Megušar et al., 2023). Non-affinity purification of RNA of different sizes can be performed with multimodal monolith weak anion-exchanging properties and an isoelectric point of 5.3 (CIM Swiper) under near-neutral conditions (Miklavčič et al., 2023) — the IVT mixture is loaded in 50 mM Na-citrate, pH 5.0, to achieve selective binding of DNA and RNA (NTPs and other IVT components elute in flow-through). In agreement with previously reported work on mRNA, a peak with CIMac PrimaS profile consistent with the DNA template or short RNA fragments was detected in the 0.4 M NaCl wash (pH 5.0). The tRNA eluted when the pH was increased to 7.5 (Figures 4A, B). However, the overall purity was lower than observed for gel-excised tRNA; residual RNA fragments could be detected in the elution fraction when separated on a PAGE gel, particularly at higher loadings (Figure 4C).

**FIGURE 5:**
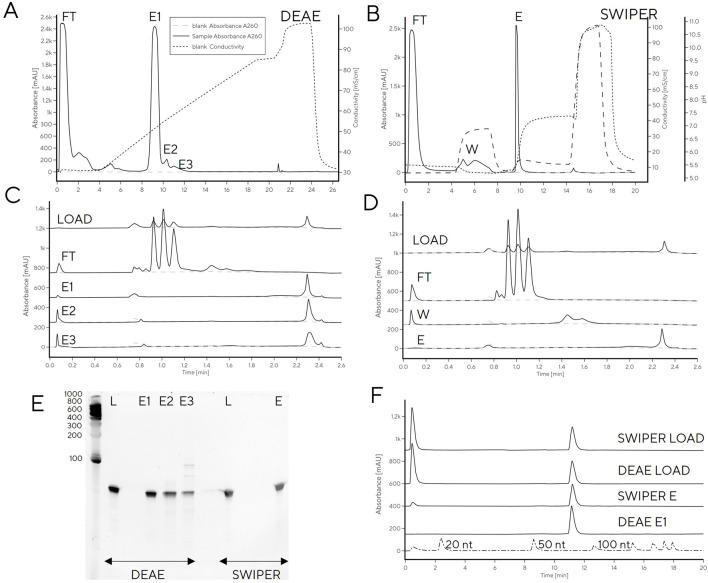
tRNA purification with **(A)** Swiper and **(B)** DEAE. CIMac PrimaS analytics of collected fractions from **(C)** Swiper: load, FT, Wash, Elution; and **(D)** Swiper: load, FT, E1, E2, and E3. **(E)** Fractions resolved on 10% TBE-Urea gel (left) and **(F)** IP-RP analysis of DEAE/Swiper elution fractions.

In order to remove low-level contamination of the elution fraction, the Swiper wash step could potentially be further optimized (e.g., wash salt concentration increased). However, this risked decreasing the recovery of pure tRNA in the elution fraction [modulating wash concentration of NaCl on PrimaS (Megušar et al., 2023)]. Instead, we explored tRNA binding to weak anion exchange column (CIM DEAE) to increase selectivity between DNA and tRNA by virtue of stronger anion exchange character compared to Swiper. A head-to-head comparison of Swiper and DEAE revealed that the impurities which bound to Swiper and were partially removed in the wash step (e.g., characteristic CIMac PrimaS peak with RT 1.4 min, Figures 5A, C), did not bind to DEAE (Figures 5B, D)

While recovery of the main elution fraction was higher for Swiper (>90%) than DEAE (85%) (Supplementary Table S3), DEAE resulted in higher purity, presumably due to its stronger anion exchange character which can exploit minor charge differences between tRNA and fragments for separation (Figures 5E, F). In particular, PAGE gel indicated high purity of DEAE E1, and detected RNA fragments in fractions E2 and E3. The purity of DEAE E1 was confirmed by IP-RP HPLC which detected a single peak at target molecular size. In contrast to DEAE E1 elution, Swiper E1 elution contained shorter fragments detectable by IP-RP (peak at RT 0.5 min) and a smear observed in PAGE gel (Figure 5E, F).

**FIGURE 6:**
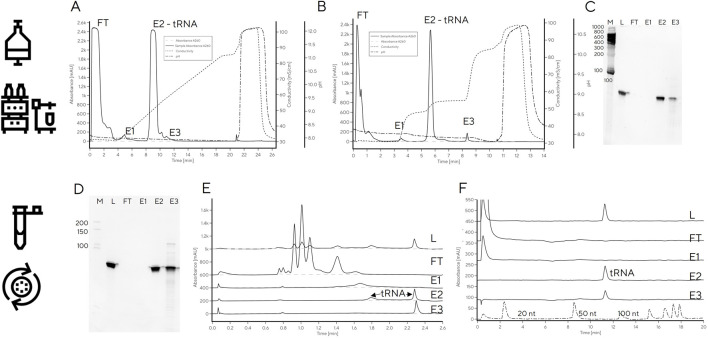
tRNA purification with step elution with CIM DEAE using HPLC **(A–C)** or centrifuge **(D–E)**. **(A)** Linear salt gradient elution of IVT mixture containing tRNA on CIM DEAE operated with HPLC; **(B)** step elution of IVT mixture containing tRNA on CIM DEAE operated with HPLC. **(C)** PAGE gel of step elution fractions FT, E1-E3. **(D)** PAGE gel of step elution fractions of IVT mixture containing tRNA on CIM DEAE operated with centrifuge. **(E)** CIMac PrimaS analytics of CIM DEAE spin column elution fractions (LOAD, FT, E1, E2, and E3). **(F)** RP-IP analysis of CIM DEAE spin column elution fractions.

Finally, the linear gradient elution on DEAE was converted to a three-step elution based on the conductivities required for elution of each target fraction (48, 54 and 86 mS/cm for E1, E2 and E3, respectively, Figures 6A, B). Due to the large differences in conductivity required for elution of each fraction, the separation was easily converted to a step elution with predicted purity profiles. Application of a step elution approach using an HPLC system was successful; the main tRNA fraction, E2, was pure and free of RNA fragments and DNA template which were removed in fraction E3 (Figure 6C), consistent with previous reports on Mono Q which showed DNA template elution after tRNA (Koubek et al., 2013). Binding capacity was determined from breakthrough curve and from elution, both resulting in binding capacity of 3.1 mg/mL (Supplementary Figure S4).

To facilitate tRNA purification in the absence of a HPLC system, the step elution method was transferred to spin purification columns containing a DEAE monolith disc. Centrifugal force was used for convective mass transfer of the tRNA-containing sample through the monolith stationary phase housed in conical tubes. The main elution (E2) contained highly pure tRNA (Figures 6D–F) with 90% recovery, as determined by UV. This is important, because it significantly shortens the purification time required to obtain highly pure tRNA compared to traditional gel excision and avoids the need for using toxic denaturants (e.g., acrylamide in preparation of PAGE gels) without a loss in recovery.

### Functional testing of synthetic tRNAs

tRNA constructs for all three tRNA tested (INI, ELO2 and ELO3) were all successfully synthesised using our optimized IVT protocol and purified with CIM DEAE using HPLC (Supplementary Figure S5). A second sample of INI was prepared and purified by SWIPER for comparison. Interestingly, DEAE elution peaks of ELO2 and ELO3, but not INI, contained a shoulder. These were identified as N+1/N+2 products by PAGE (Supplementary Figure S5D). Single nucleotide resolution of oligonucleotide mixtures has previously been shown on CIM DEAE (Podgornik et al., 1999), but not on construct sizes of >70 nucleotides, which we demonstrate here.

For use in codon reprogramming for mRNA display applications, it is necessary for the tRNA to be aminoacylated before addition to a cell-free translation system. One commonly used aminoacylation method is mediated by short RNA ribozymes called Flexizymes (Goto et al., 2011b; Murakami et al., 2003; Murakami et al., 2006; Niwa et al., 2009; Passioura and Suga, 2013). To demonstrate that the tRNA produced and purified under our optimised conditions could be aminoacylated and processed by the ribosome, we chose two unnatural amino acids to incorporate into a short peptide sequence. The initiator tRNA (INI) were loaded with *N*-biotinylated-l-phenylalanine, while the elongator tRNAs (ELO2, ELO3) were loaded with d-phenylalanine (Sako et al., 2008; Suga et al., 2011). By omitting methionine from the translation mixture and adding aminoacylated tRNAs, we were able to reprogram the initiator and elongator methionine codons to the noncanonical amino acids.

**FIGURE 7:**
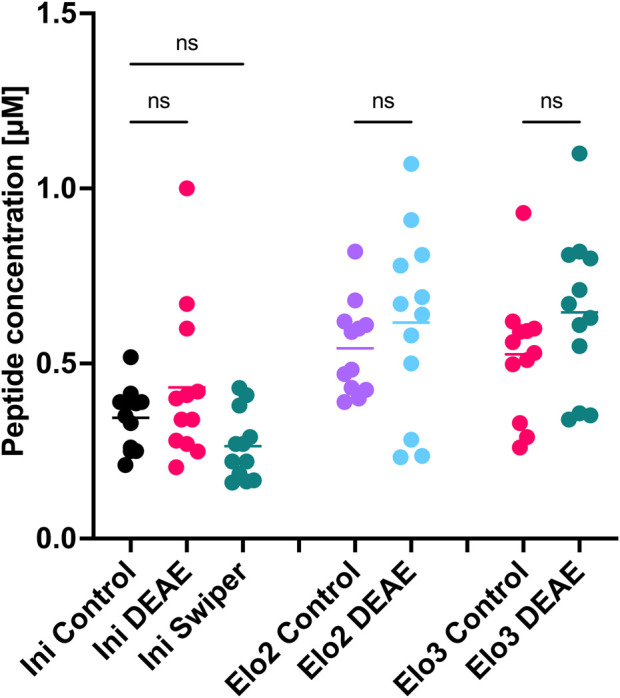
Comparison of tRNA aminoacylation and *in vitro* translation efficiencies. Assay performed as technical triplicates within independent triplicates. Ordinary one-way ANOVA followed by Šídák’s multiple comparisons test was performed using GraphPad Prism version 10.2.3 for MacOS, GraphPad Software, Boston, Massachusetts USA, www.graphpad.com.

To compare the different batches of tRNA, we carried out cell-free *in vitro* translation of an mRNA template encoding a HiBit sequence. Translated peptides can be characterised through MALDI-TOF mass spectroscopy and quantified using a HiBit luminescence assay (Chan et al., 2023; Sato et al., 2022). MALDI-TOF MS confirmed that the correct sequence had been translated and the ribosome had incorporated the unnatural amino acids, loaded onto the tRNA, into the peptide sequence suggesting our synthetic tRNAs were functional (Supplementary Figure S6). Comparison of translation yields by HiBit assay showed no statistically significant variation in the yields from translations using tRNAs prepared by the different methods (Figure 7). This demonstrates that the tRNAs produced and purified through our optimised conditions can be aminoacylated and are functionally indistinguishable to the ribosome from those produced with GMP and purified on PAGE gel.

## Conclusion

Through at-line HPLC monitoring of IVT reactions, we first optimized tRNA production method to achieve the highest reported tRNA yield (4.5 g/L), primarily by increasing GTP concentration which we showed to be the limiting factor. tRNA was produced in <4.5 h, while incubation overnight led to a minor increase in yield (10% for high-producing reactions). An efficient purification of tRNA was achieved with CIM DEAE, either coupled to a chromatography system operated in linear gradient or step elution mode or with a spin column, both achieving 90% recovery.

The whole process of production and purification of tRNA was shortened from approximately a day and a half to 6 h. tRNAs produced by the optimized IVT procedure were shown to be functionally indistinguishable from tRNA produced by a traditional method employing GMP in test *in vitro* translation reactions.

This study demonstrated how at-line HPLC monitoring can be used to monitor, and improve, production yields of IVT-derived tRNAs. Though the IVT monitoring approach is now widely used for mRNA applications, we show that adjustments in HPLC peak interpretation are required to apply the methodology to tRNA. With the advent of CRISPR-Cas technologies, which require both mRNA and guide RNAs of different lengths, the ability of at-line monitoring techniques to adapt to target RNA length will be of great importance. Furthermore, we show that multimodal purification optimized for mRNA and saRNA are applicable but surpassed in resolution and purity by traditional weak AEX monolith ligands (DEAE) with binding capacity comparable to mRNA (3 mg/mL). Due to a significantly lower overall negative charge of tRNA compared to mRNA due to shorter length, recovery from AEX is high while its high resolution is particularly useful for tRNA when separation of N+1/N+2 products is required. To our knowledge, this is the first reported use of spin columns for rapid isolation of tRNA, which can significantly ease efforts to produce tRNA for laboratory, pre-clinical and potentially clinical use. We foresee the use of this methodology for production of tRNA for *in vitro* translation studies, pre-clinical development of tRNA therapies, as well as for structural studies (NMR, X-ray, cryo-EM) of other short RNA molecules produced by IVT, including riboswitches, ribozymes and pre-micro RNAs.

## Figures and Tables

**Table:** 

**Abbreviations**	
**AGE**	Agarose gel electrophoresis
**BSA**	Bovine serum albumin
**BTP**	Bis-tris propane
**CAPS**	N-cyclohexyl-3-aminopropanesulfonic acid
**CIM**	Convective Interaction Media
**CIP**	Cleaning-in-place
**circRNA**	Circular RNA
**CMC**	Chemistry, manufacturing and control
**CoG**	Cost of Goods
**CV**	Column volume
**DS**	Drug Substance
**FLD**	Fluorescence detection
**FPLC**	Fast protein liquid chromatography
**Gu-HCl**	Guanidine hydrochloride
**HSW**	High-salt wash
**IVT**	*in vitro* transcription
**LNP**	Lipid nanoparticles
**mAb**	Monoclonal antibody
**MP**	Mobile phase
**mRNA**	Messenger ribonucleic acid
**MWCO**	Molecular weight cut-off
**NTP**	Nucleotide triphosphate
**Oligo dT**	Oligo deoxythymidylic acid
**polyA**	Poly-adenine
**pDNA**	Plasmid DNA
**RT**	Retention time
**saRNA**	Self amplifying RNA
**ssRNA**	Single-stranded RNA
**TFF**	Tangential flow filtration
**TEAA**	Triethylammonium acetate

## Data Availability

The raw data supporting the conclusions of this article will be made available by the authors, without undue reservation.
