# Corrigendum: HPLC for at-line reaction monitoring and purification improves yield and purity of tRNA

**DOI:** 10.3389/fmolb.2024.1507191

**Published:** 2024-10-22

**Authors:** Polona Megušar, Ewen D. D. Calder, Tina Vodopivec Seravalli, Sergeja Lebar, Louise J. Walport, Rok Sekirnik

**Affiliations:** ^1^ Sartorius BIA Separations d.o.o., Ajdovščina, Slovenia; ^2^ Department of Chemistry, Molecular Sciences Research Hub, Imperial College London, London, United Kingdom; ^3^ Protein-Protein Interaction Laboratory, The Francis Crick Institute, London, United Kingdom

**Keywords:** tRNA, *in vitro* transcription, HPLC, chromatography, anion exchange

In the published article, there was an error. Incorrect chromatographic system supplier was named in the Methods section.

A correction has been made to **Materials and methods**, *Purification development*, Paragraph 1. This sentence previously stated:

“Chromatographic purification was performed on an HPLC system (Knauer, Berlin, DE) with four pumps and a multiwavelength UV-Vis detector (10 mm flow cell path length). Patfix software (Sartorius BIA Separations) was used for instrument control and data acquisition.”

The corrected sentence appears below:

“Chromatographic purification was performed using PATfix system (Sartorius BIA Separations, Slovenia) equipped with quaternary pump and a multiwavelength UV-Vis detector (10 mm flow cell path length). PATfix 2.0 software (Sartorius BIA Separations) was used for instrument control and data acquisition.”

The authors apologize for this error and state that this does not change the scientific conclusions of the article in any way. The original article has been updated.

