# Supplementary material for: HPLC for at-line reaction monitoring and purification improves yield and purity of tRNA
Source: Front Mol Biosci. 2024 Sep 27;11:1443917. doi: 10.3389/fmolb.2024.1443917 (PMC11466894; doi:10.3389/fmolb.2024.1443917)
Supplement: Supplementary file 1 [file DataSheet1.docx]

HPLC for at-line reaction monitoring and purification improves yield and purity of tRNA

Polona Megušar^1^†, Ewen D.D. Calder^2,3^†, Tina Vodopivec Seravalli^1^, Sergeja Lebar^1^, Louise J. Walport^2,3,*^, Rok Sekirnik^1,*^

^1^ Sartorius BIA Separations d.o.o., Mirce 21, SI-5270 Ajdovščina, Slovenia

^2^ Department of Chemistry, Molecular Sciences Research Hub, Imperial College London, London W12 0BZ, United Kingdom

^3^Protein-Protein Interaction Laboratory, The Francis Crick Institute, London NW1 1AT, United Kingdom

‡ Authors contributed equally

*Correspondence should be addressed to the following authors:

Louise Walport, Department of Chemistry, Molecular Sciences Research Hub, Imperial College London, London W12 0BZ, United Kingdom, l.walport@imperial.ac.uk

Rok Sekirnik, Sartorius BIA Separations d.o.o., Mirce 21, SI-5270 Ajdovščina, Slovenia, rok.sekirnik@biaseparations.com

**Keywords:** tRNA; in vitro transcription; HPLC; chromatography; anion exchange

**Supplementary Information**

**Supplementary Methods**

General experimental

DNA primers for PCR were purchased from IDT. DTT, MgCl_2_, Spermidine, Triton X-100 and CaCl_2_, urea and acrylamide solutions were from Merck Life Sciences, T7 RNA polymerase and DNase from Thermo Fisher Scientific, NTPs were from Jena Biosciences or Mebep Bioscience, China. Reagents for chemical synthesis were from either Fluorochem or Merck Life Sciences and were used without further purification. NMR was carried out on a 400 MHz Bruker AV instrument and were referenced to published residual solvent signals (Babij et al., 2016). LC-MS monitoring of reactions used to synthesise unnatural amino acids was carried out on a Waters Acquity UPLC H-Class PLUS system with a C18 column (Aquity UPLC BEH C18, 2.1x50 mm, 1.7 µm), running a 5–98% linear gradient of water and acetonitrile with 0.01% formic acid over 4 minutes.

Preparation of DNA of eFx, tRNA and mRNA

Preparation of DNA templates containing a T7 promoter to produce initiator and elongator tRNAs, and mRNA for *in vitro* translation were produced by two rounds of overlapping PCR according to previously described protocols (Goto et al., 2011a; Goto et al., 2011a; Iwane et al., 2021). DNA was purified by phenol/chloroform extraction and ethanol precipitation and was then used without further purification in IVT reactions. tRNA sequences used in this study are given in Supplementary Table 1.

Reference protocol for *in vitro* transcription of eFx and mRNA

The corresponding DNA template was incubated under the conditions described in Supplementary Table 2 at 37 °C for 16–24 h. T7 Buffer is comprised of 400 mM Tris (pH 8.0), 10 mM Spermidine and 0.1% (v/v) Triton X-100.

The solution was adjusted by the addition of 0.1 v/v of DNase buffer (100 mM Tris-HCl (pH 7.5), 25 mM MgCl_2_ and 5 mM CaCl_2_) and treated with 30 U/mL of DNase I (RNase free) at 37 °C for 1 h. The enzymes were inactivated by addition of 0.5 M EDTA (pH 8.0), and the RNA precipitated with isopropyl alcohol.

PAGE purification of eFx and control tRNAs

eFx was purified by denaturing PAGE using a 6 M Urea, 12% acrylamide gel while tRNAs were purified using a 4 M Urea, 8% acrylamide gel, both types of gel were 15 × 15 cm × 2 mm and electrophoresis was carried out at 230 V for 60 mins. RNA was visualized in-gel using a handheld UV lamp at 265 nm, excised, and extracted twice with 0.3 M sodium acetate solution, then ethanol precipitated.

Chemical synthesis of d-Phe-CME.TFA

Triethylamine (0.15 mL, 1.1 mmol, 2.2 eq) was added to a solution of N-Boc-D-Phe-OH (0.13 g, 0.50 mmol, 1.0 eq) in a 1:1 mixture of acetonitrile and chloroacetonitrile (0.5 mL each) and stirred at room temperature (rt) for 18 h. The reaction was monitored by LC/MS and when complete the solution was concentrated *in vacuo*. The residue was redissolved in dichloromethane (0.25 mL) and trifluoroacetic acid (0.25 mL) was carefully added. The reaction was stirred at rt for 1 h. Toluene (1 mL) was added to the solution, and it was concentrated *in vacuo*. Purification by RPHPLC (Water/MeCN+0.1% TFA) yielded the title compound as a colourless solid (21 mg, 20% over 2 steps). ^1^H NMR (400 MHz, D6-DMSO) δ_H_ = 8.67 (br.s, 3H), 7.37 – 7.28 (m, 3H), 7.27 – 7.22 (m, 2H), 5.08 (ABq, Dd_AB_ = 0.02, *J*=16.0, 2H), 4.44 (dd, *J*=7.5, 6.1, 1H), 3.17 (dd, *J*=14.1, 6.1, 1H), 3.09 (dd, *J*=14.1, 7.5, 1H); ^13^C NMR (101 MHz, D6-DMSO) δ_c_ 168.4, 158.3 (q, ^2^*J_CF_* = 31.1 Hz), 134.3, 129.4, 128.7, 127.4, 117.2 (q, ^1^*J_CF_* = 299.6 Hz), 115.2, 53.1, 50.1, 35.9.

Chemical synthesis of Bio-l-Phe-CME

*N,N*-Diisopropylethylamine (0.10 mL, 0.59 mmol, 1.0 eq) was added to a solution of
l-phenylalanine (0.97 g, 0.59 mmol, 1.0 eq) in *N,N*-dimethylformamide (15 mL). Biotin NHS ester (0.20 g, 0.59 mmol, 1.0 eq) was added and the solution was stirred at rt for 66 h then concentrated *in vacuo*. The resulting solids were washed with an ice cold 0.1 M aqueous solution of hydrochloric acid (2 mL) followed by cold water (2 mL) then dried by vacuum transfer overnight to yield *N*-biotinyl-l-phenylalanine (0.22 g, 95%) as a colourless solid which was used without further purification. ^1^H NMR (400 MHz, DMSO-*d*_6_) δ_H_ 8.10 (1H, d, *J* 8.2), 7.95 (1H, s), 7.31 – 7.15 (5H, m), 6.37 (2H, br.s), 4.42 (1H, ddd, *J* 9.8, 8.2, 4.8), 4.30 (1H, ddd, *J* 7.7, 5.1, 1.0), 4.10 (1H, dd, *J* 7.7, 4.4), 3.09 – 2.99 (2H, m), 2.82 (2H, m), 2.58 (1H, d, *J* 12.8), 2.04 (2H, dd, *J* 7.3, 6.5), 1.75 – 1.32 (6H, m); LC/MS Rt 1.21 min, >95%, m/z (ESI^+^) 392 (100%, [M+H]^+^), (ESI^–^) 390 (100%, [M–H]^–^). Triethylamine (0.71 mL, 0.51 mmol, 2.0 eq) was added to a suspension of *N*-biotinyl-l-phenylalanine (0.10 g, 0.26 mmol, 1.0 eq) in a 1:1 mixture of acetonitrile (0.65 mL) and chloroacetonitrile (0.65 mL) and stirred at rt for 18 h. The reaction mixture was concentrated *in vacuo* to give a pale-yellow solid (97 mg). Half of this solid was dissolved in the minimal amount of a solution of 50% acetonitrile, 49 % water and 1% dimethyl sulfoxide and purified by preparative HPLC (Water/MeCN+0.1% TFA). The fractions containing product were concentrated by lyophilisation to yield the title compound (13 mg, 24% based on half purified) as a colourless solid. ^1^H NMR (400 MHz, CD_3_OD) δ_H_ 7.35 – 7.25 (2H, m), 7.27 – 7.18 (3H, m), 4.89 (2H, s), 4.73 (1H, dd, *J* 9.4, 5.7), 4.49 (1H, ddd, *J* 7.9, 5.0, 0.9), 4.27 (1H, dd, *J* 7.9, 4.5), 3.20 (1H, dd, *J* 13.9, 5.7), 3.15 (1H, ddd, *J* 8.6, 6.1, 4.4), 3.00 (1H, dd, *J* 13.9, 9.4), 2.93 (1H, dd, *J* 12.8, 5.0), 2.71 (1H, d, *J* 12.7), 2.21 (1H, ddd, *J* 14.1, 7.1, 4.5), 2.16 (1H, ddd, 14.1, 7.1, 4.2), 1.75 – 1.62 (1H, m), 1.63 – 1.47 (3H, m), 1.41 – 1.24 (2H, m); ^13^C NMR (400 MHz, CD_3_OD) δ_C_ 176.1, 171.9, 166.1, 137.8, 130.2, 129.6, 128.0, 115.9, 63.3, 61.6, 56.9, 54.9, 50.1, 41.0, 38.0, 36.2, 29.44, 29.36, 26.6; LC/MS Rt 1.20 min, >99%, m/z (ESI^+^) 431 (100%, [M+H]^+^), (ESI^–^) 475 (100%, [M+FA]^–^).

Preparation of Solution A (solA)

SolA, as used in the main text, is a solution comprising HEPES-KOH (pH 7.6, 50 mM), ATP (2 mM), GTP (2 mM), CTP (1 mM), UTP (1 mM), creatine phosphate (20 mM), potassium acetate (100 mM), spermidine (2 mM), magnesium acetate (12 mM), E. coli tRNA mix (1.5 mg/mL) and dithiothreitol (14 mM).

Preparation of samples for MALDI-TOF MS

Elute solution (80% acetonitrile, 0.5% acetic acid), Wash solution (4% acetonitrile, 0.5% acetic acid) and a half-saturated solution of α-CHCA in Elute solution were prepared. A C18-zip tip (Merck Millipore) was washed with Elute solution (15 μL) then Wash solution (15 μL). The translation mixture (3.5 μL) was loaded onto the tip. The loaded spin tip was washed with Wash solution (2 × 15 μL). The peptide was eluted from the tip with half-saturated α-CHCA in Elute solution (1.2 μL) directly onto the MALDI plate and allowed to dry in air. MALDI-TOF MS was carried out on a Shimadzu MALDI 8030 system in positive, linear mode with a laser power setting between 20 and 50, collecting 100 profiles at 5 shots, 200 Hz between 1–3 or 1–4 kDa.

HiBiT assay (Promega)

The translation mix (0.5 μL) was added to phosphate buffered saline with Tween®-20 added (0.1 M, 0.5% Tween, 250 μL). Following the manufacturer’s instructions, the diluted translation mix (1 µL) was added to the detection solution (100 µL) and shaken for 10–20 min. The luminescence was then measured (Clariostar, 240–900 nm), and the concentration of peptide in the translation mix was calculated by comparison to a standard curve.

Gel electrophoresis

tRNA integrity was analyzed with TBE-Urea PAGE gel electrophoresis (10% denaturing polyacrylamide Novex™ TBE-Urea Gel, cat. no.: EC68752BOX; Invitrogen™) run at 230 V for 75 min, using XCell SureLock Mini-Cell Electrophoresis System (Thermo Fisher Scientific), filled with 1x TBE Running buffer (89 mM Tris, 89 mM Boric acid, 2 mM EDTA). Gels were stained with SYBR™ Gold Nucleic Acid Gel stain (Invitrogen™) and visualized with UV on iBright 1500 (Thermo Fisher Scientific).

**Supplementary Figures**


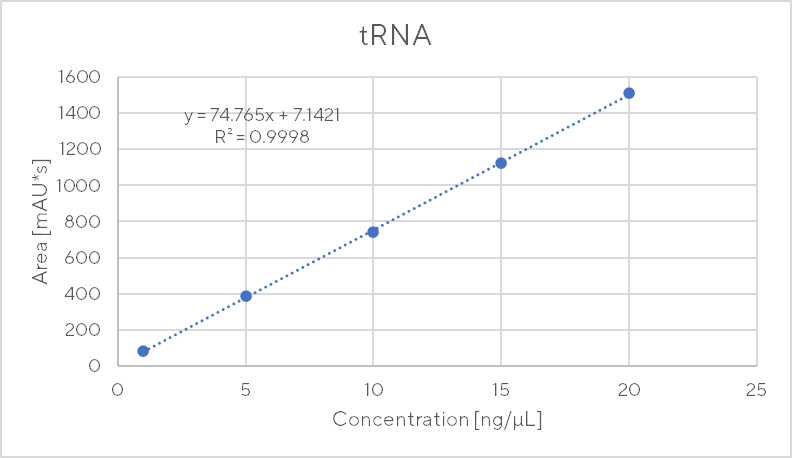


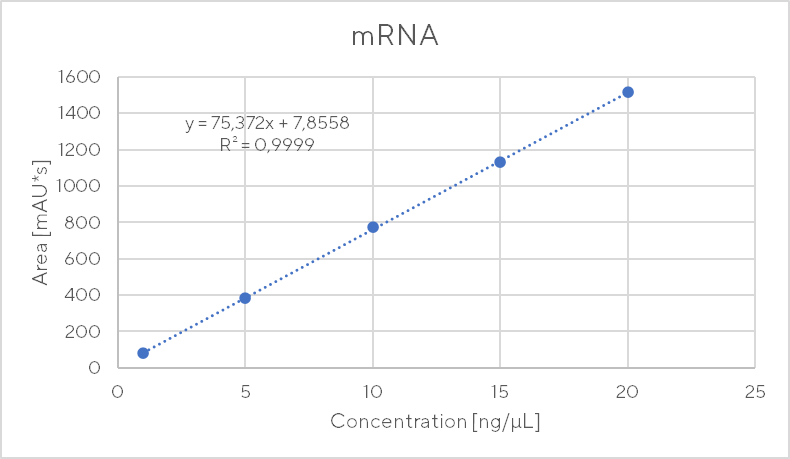


**Supplementary Figure 1: CIMac PrimaS HPLC calibration curves for quantification of tRNA (top) and mRNA (eGFP, 995 nt, bottom).**


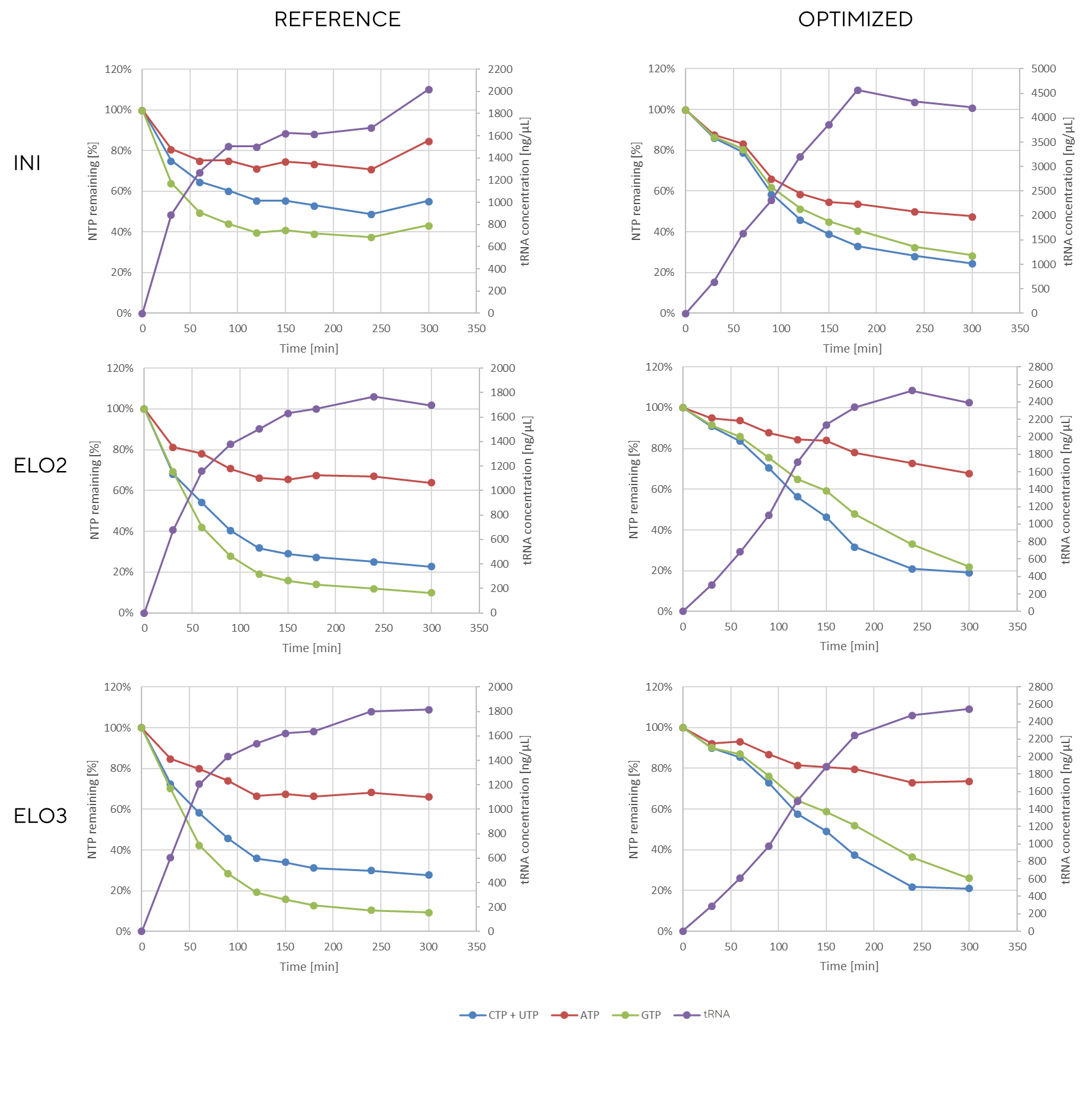


**Supplementary Figure 2: Comparison of IVT yield with same IVT protocol for three tRNA constructs. Reference IVT condition (IVT1, Table 2, main text) and protocol optimized for tRNA construct INI (IVT 6, Table 2, main text) was tested on ELO2 and ELO3.** CIMac PrimaS was used for monitoring chromatographic peaks corresponding to CTP/UTP, ATP, GTP and tRNA.


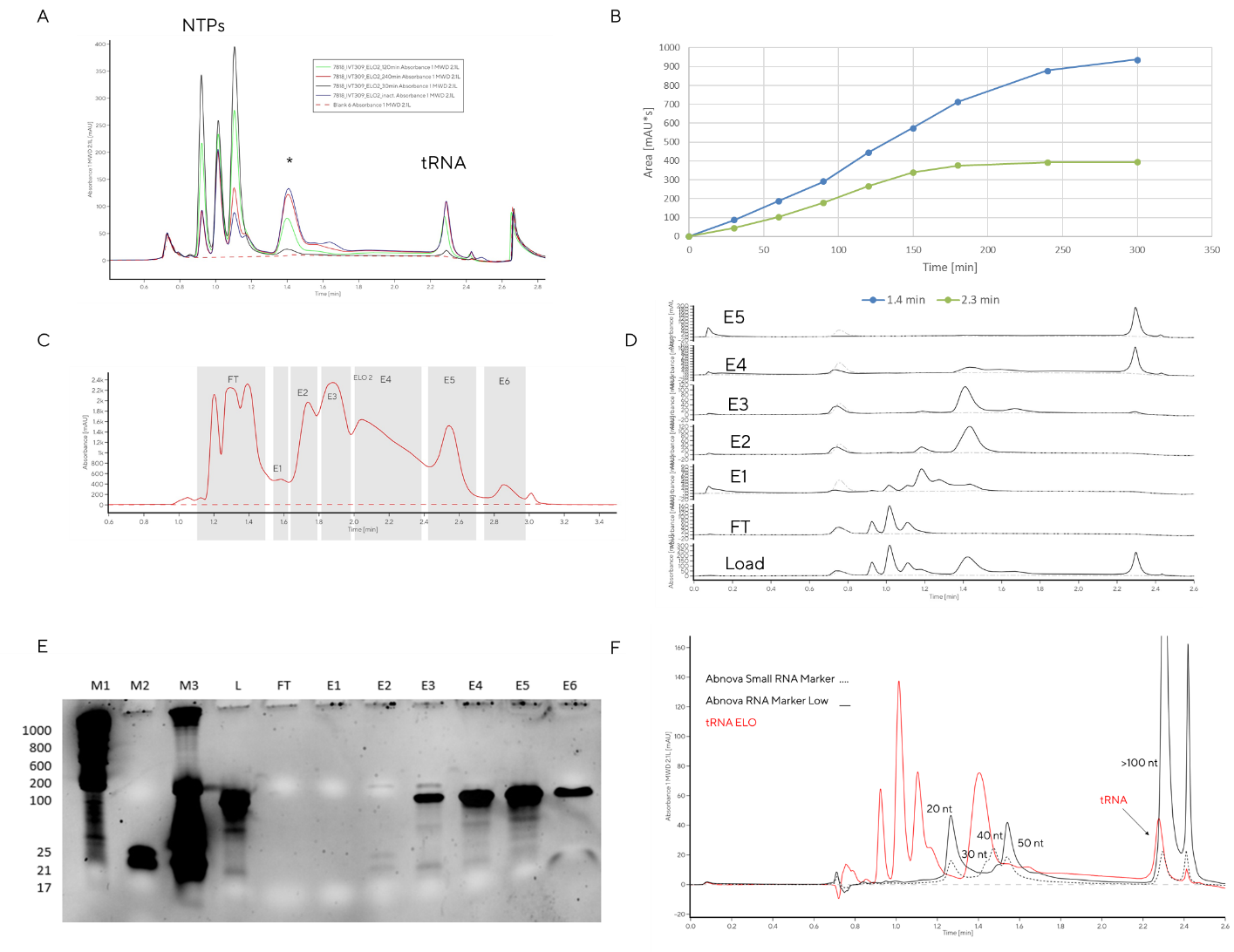


**Supplementary Figure 3: CIMac PrimaS analytical chromatograms of ELO2/ELO3 identify increase in chromatographic peak at 1.4 min.** A) CIMac PrimaS analytical chromatograms of IVT at-line monitoring (time-points 30 min, 120 min, 240 min and inactivated sample). B) Peak area of 1.4 min increases faster than peak at 2.3 min (tRNA). C) 50 µg loading of ELO2 onto CIMac PrimaS for peak-collection. D) CIMac PrimaS profiles of elution fractions. E) PAGE of elution fractions reveal the presence of 20-30 mer RNA species in E2, corresponding to analytical peak at 1.4 min. M1: RiboRuler Low Range RNA Ladder (Thermo Fisher), M2: microRNA Marker (NEB), M3: Small RNA Marker (Abnova). F) CIMac PrimaS overlay of ELO IVT with RNA ladders (‘small RNA marker (Abnova) and RNA marker low (Abnova) confirming molecular size of ~30 nt.


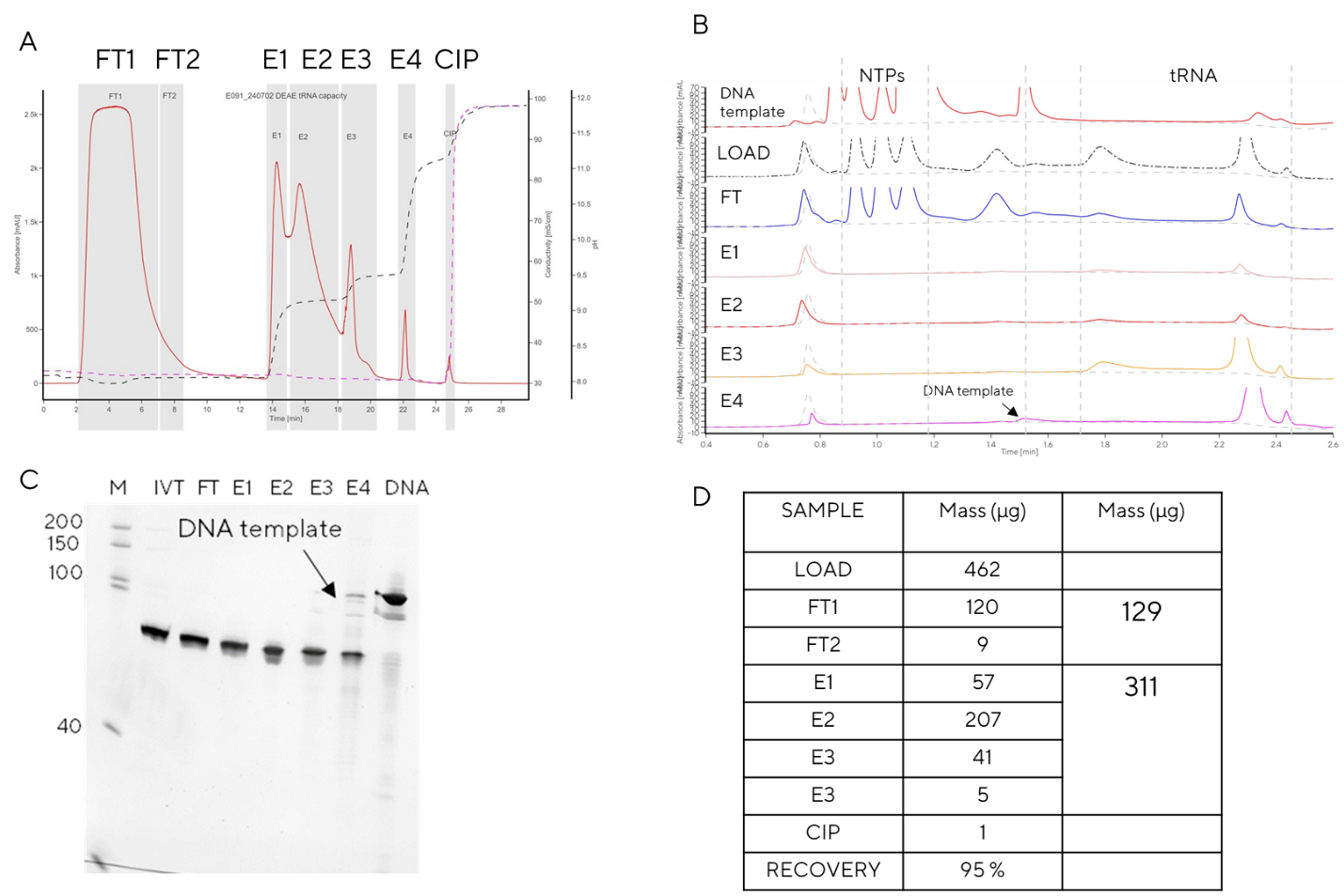


**Supplementary Figure 4: Binding capacity study of CIM DEAE for tRNA.** A) IVT mixture containing 462 µg of tRNA was loaded onto CIM DEAE disc (0.1 mL) under binding conditions. After break-through was achieved (indicated by tRNA detected in flow-through, FT), tRNA was eluted in a series of elution steps (E1-E4). Column was regenerated with CIP procedure. B) CIMac PrimaS analytical chromatograms of load, FT and elution fractions show the presence of NTPs in load and flow-through, but not in elution fractions. C) PAGE shows the purity of tRNA in elution fractions E1-E3 and co-elution of DNA template in E4. DNA template was analyzed as a sample to confirm electrophoretic profile. D) Mass-balance table of load and elution fractions.


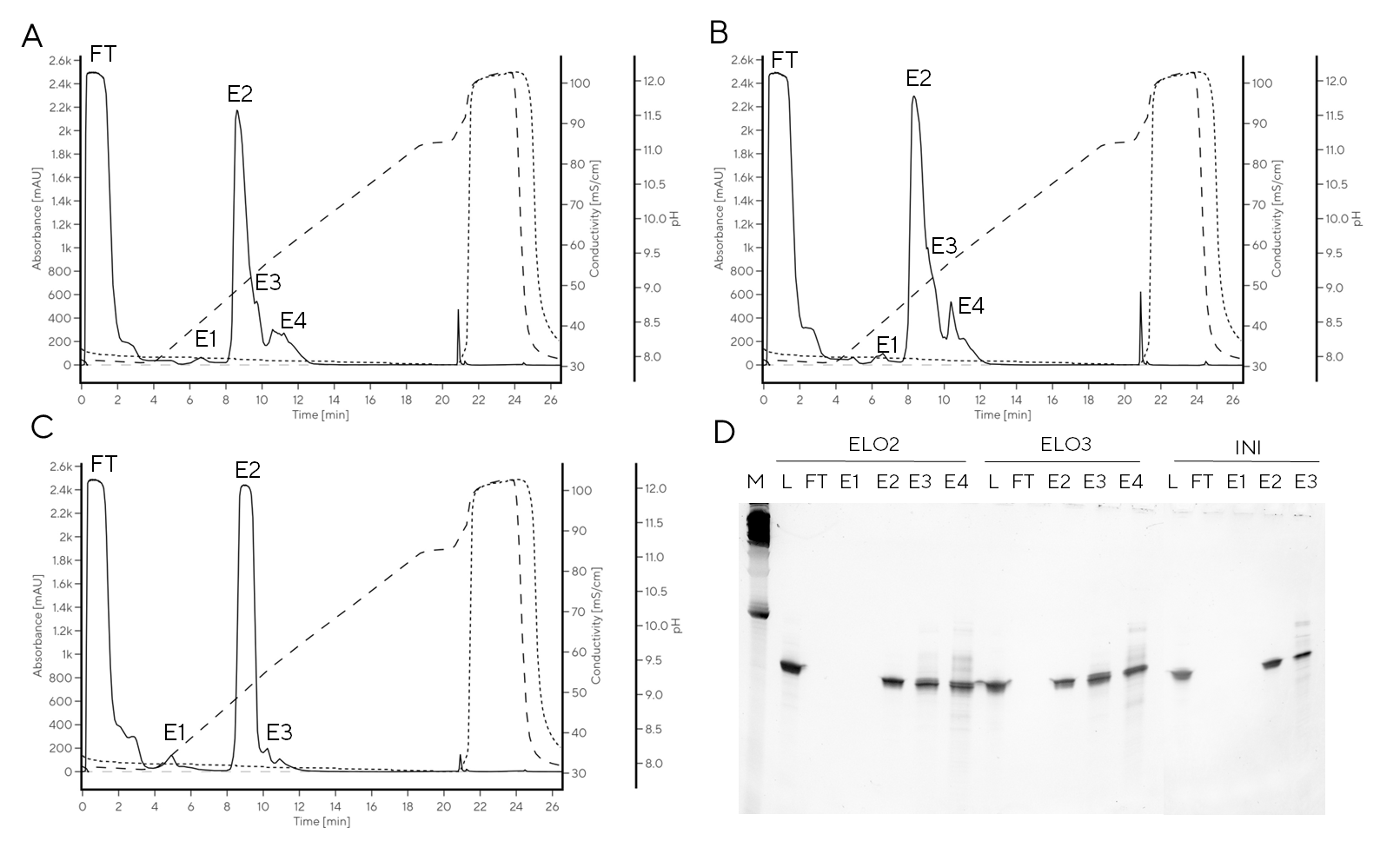


**Supplementary Figure 5: Purification of tRNA constructs with CIM DEAE operated in linear gradient mode.** A) ELO2, B) ELO3, C) INI. D) 10 % TBE-Urea PAGE gel of DEAE elution fractions for the three tRNA constructs.

INI Control:


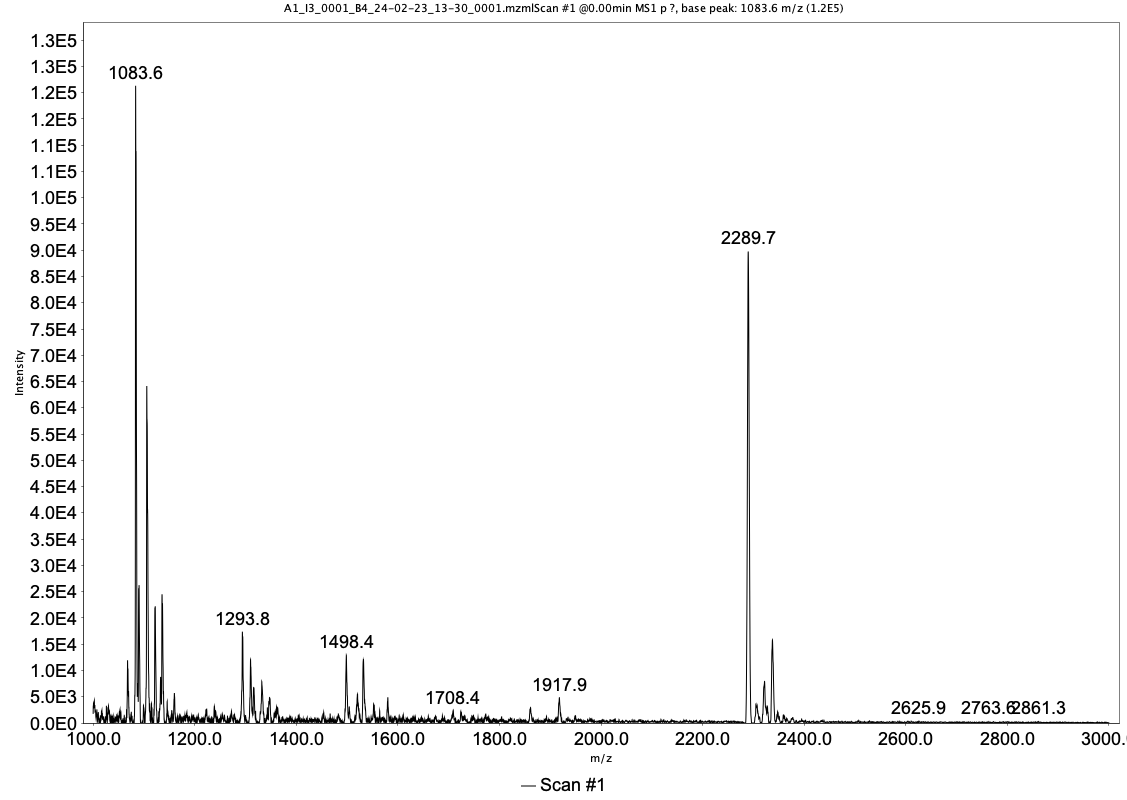


INI DEAE:


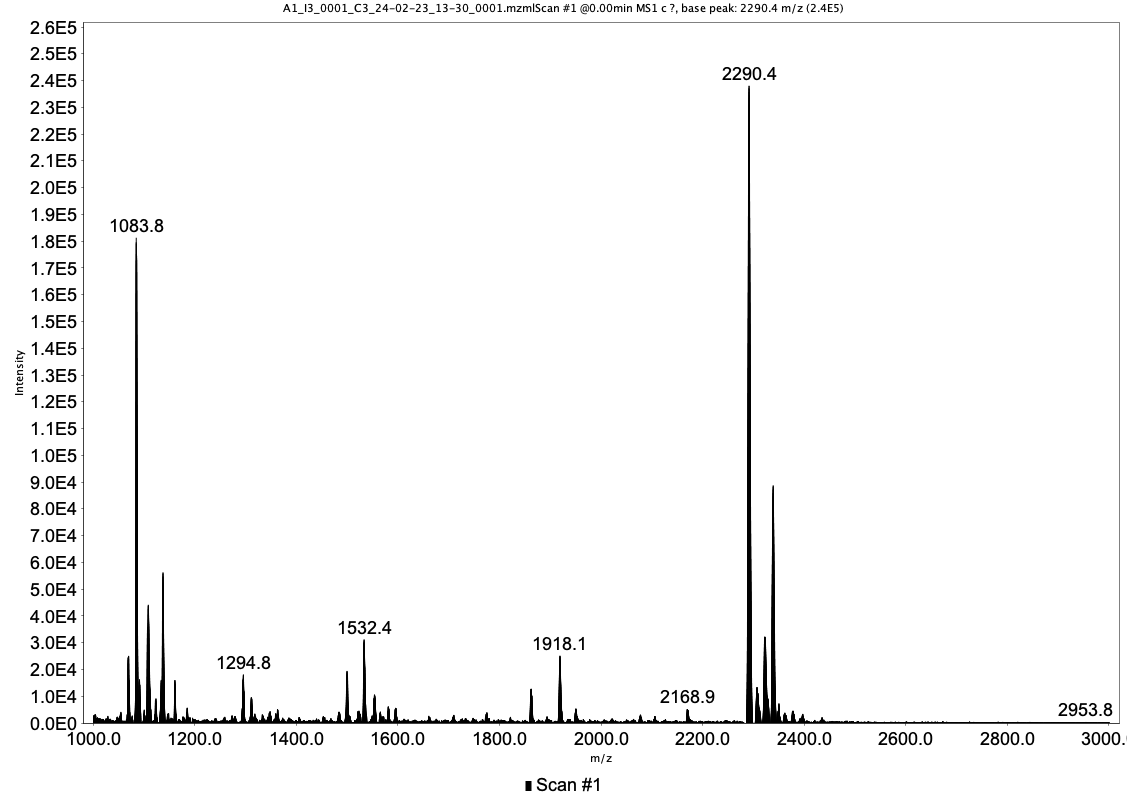


INI Swiper:


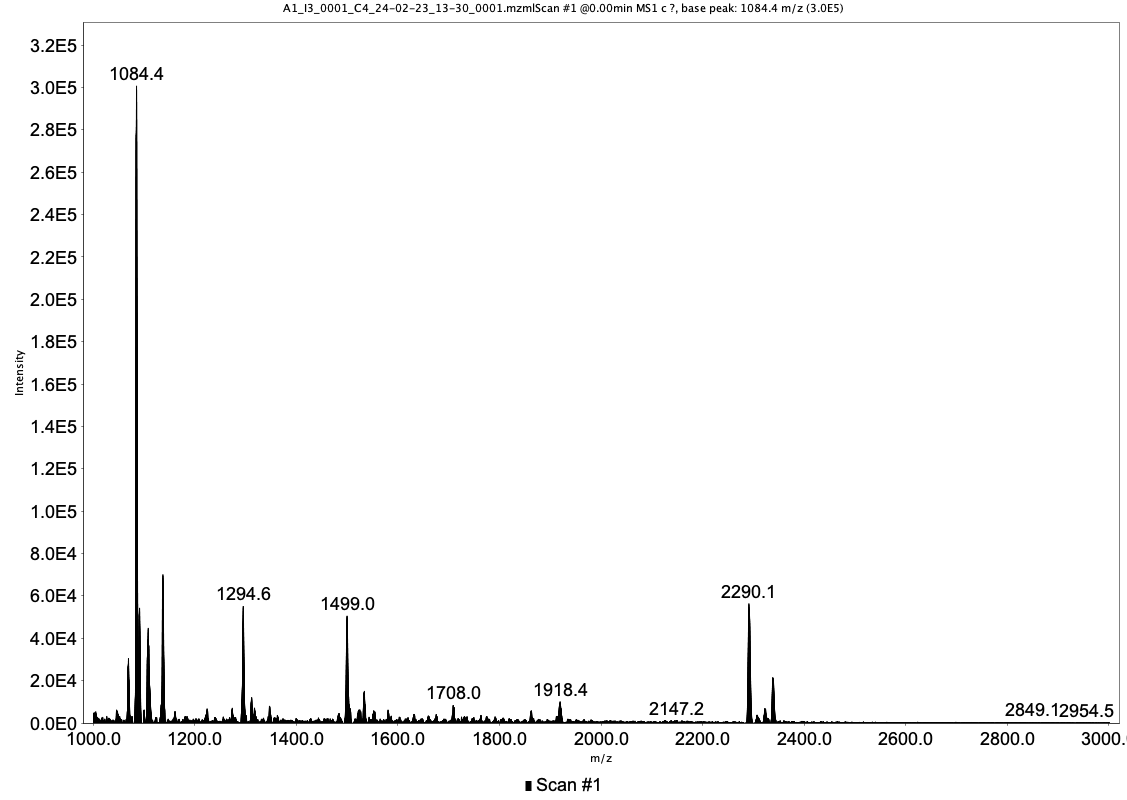


ELO2 control:


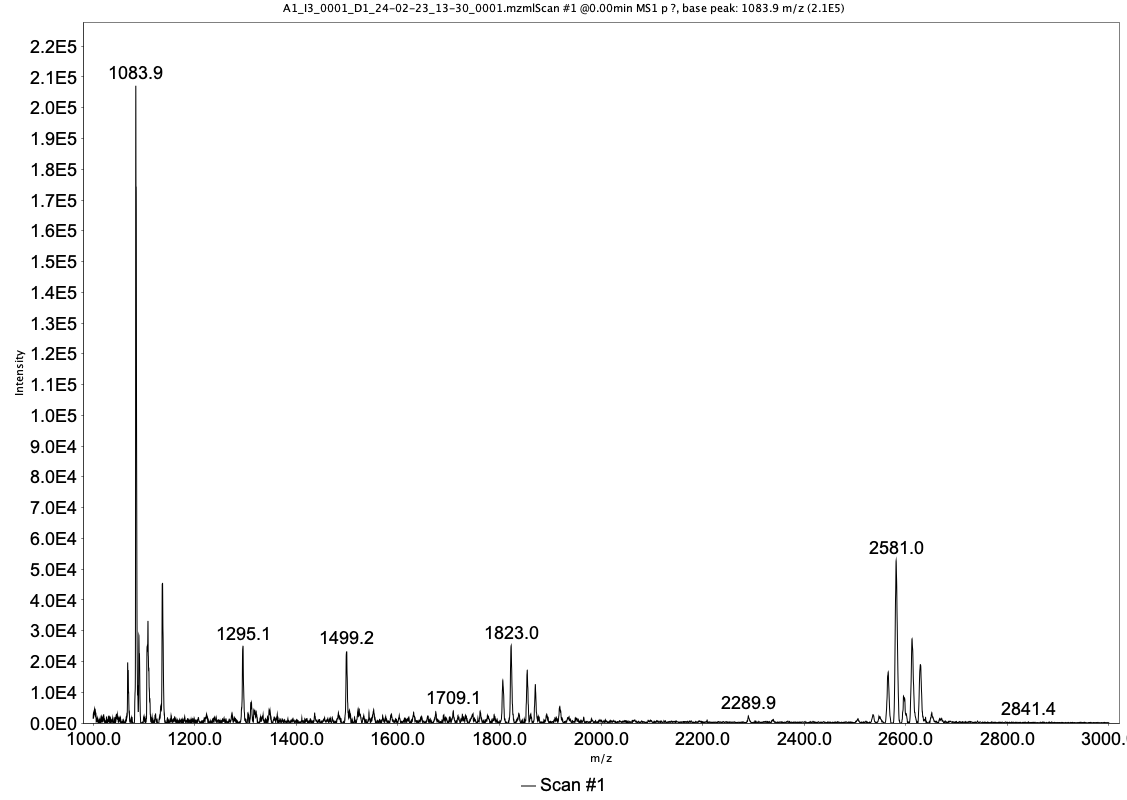


ELO2 DEAE:


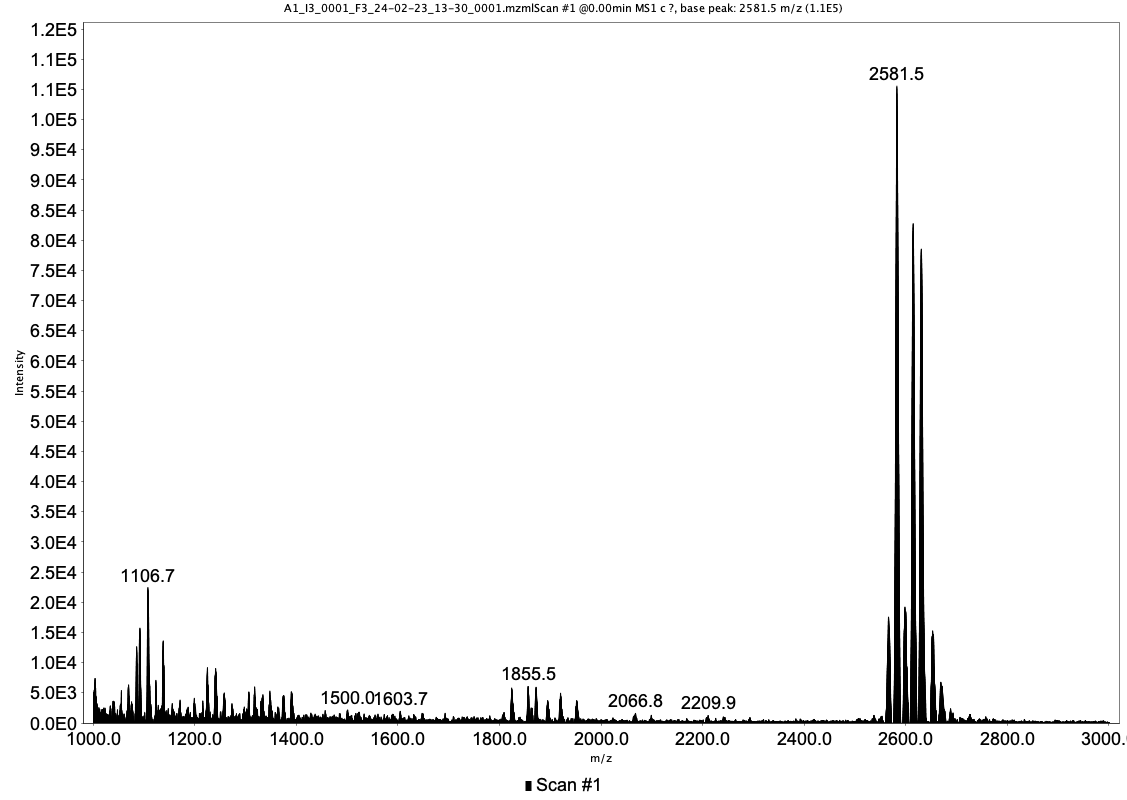


ELO3 Control:


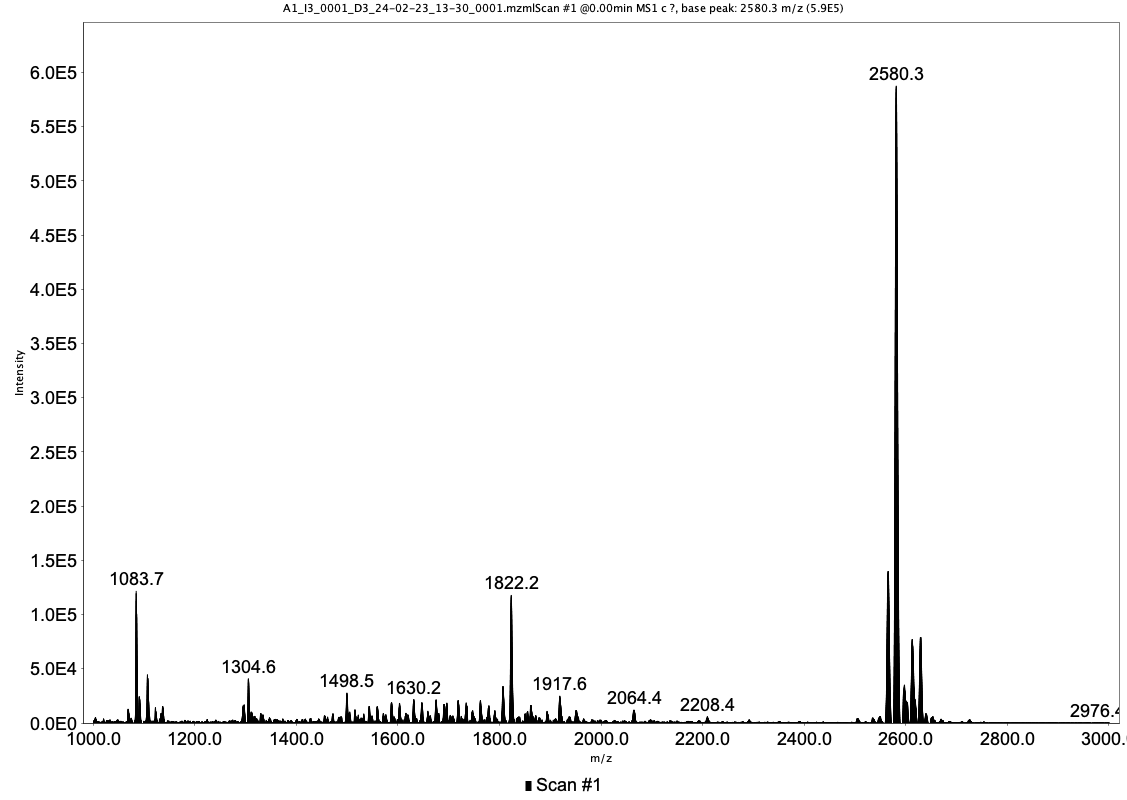


ELO3 DEAE:


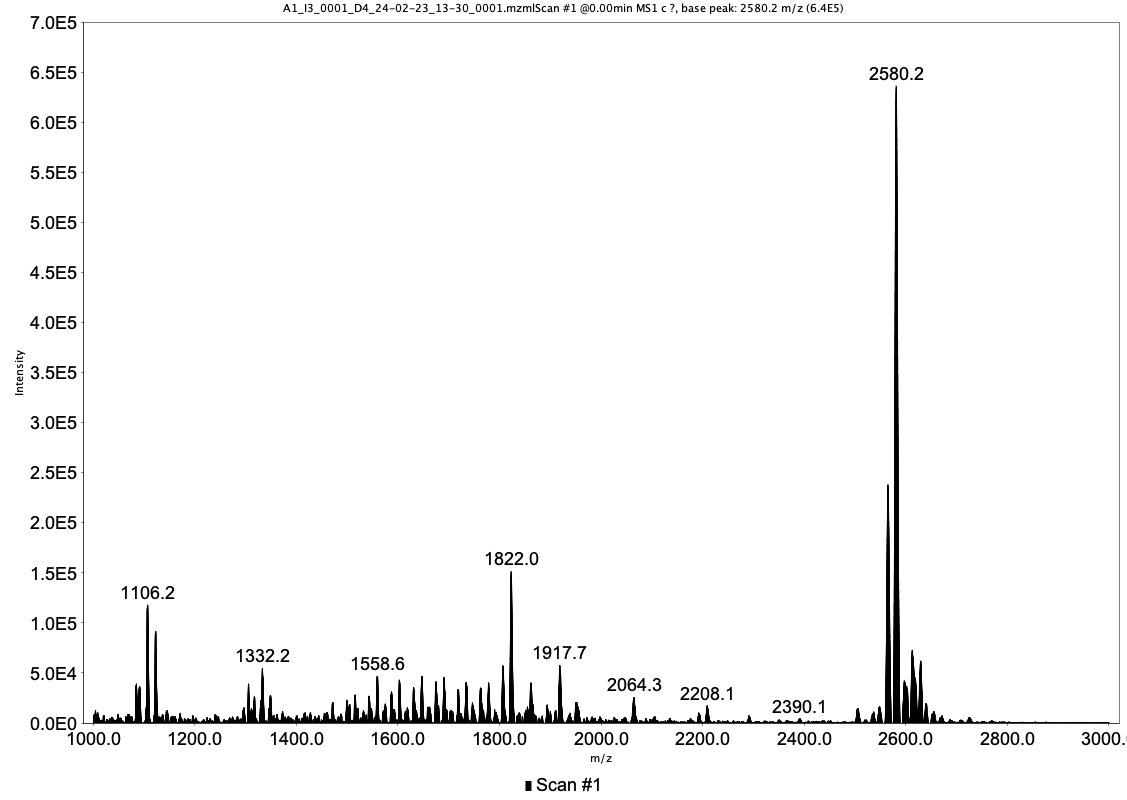


**Supplementary Figure 6: MALDI-TOF mass spectra of *in vitro* translated peptides.**

Translation of MGSVSGWRLFKKISGSGSGS with Biotin-l-Phe loaded on initiator (tRNA^fMet^_CAU_).

Translation of MGSMGSVSGWRLFKKISGSGSGS with Biotin-l-Phe loaded on initiator (tRNA^fMet^_CAU_) and d-Phe loaded on ELO2 or ELO3 (tRNA^AsnE2^_CAU_ or tRNA^AsnE3^_CAU)._

| Expected Peptide Sequence | Molecular Formula | Calculated [M+H]^+^ | Calculated [M+H_3_O]^+^ |
| --- | --- | --- | --- |
| **BiotinF**GSVSGWRLFKKISGSGSGS | C_102_H_156_N_28_O_29_S | 2271.6 | 2289.6 |
| **BiotinF**GS**d**GSVSGWRLFKKISGSGSGS | C_116_H_173_N_31_O_33_S | 2562.9 | 2580.9 |

All translations were performed in triplicate and yielded peptide masses of [M+H_3_O]^+^ within reasonable error for MALDI-TOF, a representative spectra of each translation is shown above.

**Supplementary Tables**

**Supplementary Table 1: tRNA sequences used in the study**

| tRNA | Sequence |  |
| --- | --- | --- |
| INI | GGCGGGGUGGAGCAGCCUGGUAGCUCGUCGGGCUCAUAACCCGAAGAUCG  UCGGUUCAAAUCCGGCCCCCGCAACCA | G 32.5 %  C 32.5 %  U 15.6 %  A 19.4 % |
| ELO2 | GGCUCUGUAGUUCAGUCGGUAGAACGGCGGAUUCAUAUUCCGUAUGUCA  CUGGUUCGAGUCCAGUCAGAGCCGCCA | G 28.9 %  C 25.0 %  U 26.3 %  A 19.7 % |
| ELO3 | GGCUCUGUAGUUCAGUCGGUAGAACGGCGGAUUCAUAUUCCGUAUGUCA  GGGGUUCGAGUCCCCUCAGAGCCGCCA | G 30.3 %  C 26.3. %  U 25.0 %  A 18.4 % |

**Supplementary Table 2: Reference IVT reaction recipe for eFx and mRNA production.**

| Stock | eFx | mRNA |
| --- | --- | --- |
| T7 Buffer (10×) | 1× | 1× |
| DTT (100 mM) | 10.0 mM | 10.0 mM |
| MgCl_2_ (250 mM) | 30.0 mM | 20 mM |
| NTPs (25 mM each) | 5.00 mM | 3.75 mM |
| T7 RNA Polymerase (30 U/μL) | 0.9 U/μL | 0.9 U/μL |

**Supplementary Table 3: Recovery calculation for tRNA purification with CIM Swiper and CIM DEAE.**

| FractionR | Swiper (%) | DEAE(%) |
| --- | --- | --- |
| FT | <1% | <1% |
| W | <1% | n.a. |
| E1 | 100 % | 85 % |
| E2 | n.a. | 5 % |
| E3 | n.a. | 1 % |

**References**

Goto Y, Goto Y, Katoh T, Suga H. 2011a. Preparation of materials for flexizyme reactions and genetic code reprogramming. *Protocol Exchange*. https://www.nature.com/protocolexchange/protocols/2020.

Goto Y, Katoh T, Suga H. 2011b. Flexizymes for genetic code reprogramming. *Nat Protoc* **6**:779–790.

Iwane Y, Kimura H, Katoh T, Suga H. 2021. Uniform affinity-tuning of N-methyl-aminoacyl-tRNAs to EF-Tu enhances their multiple incorporation. *Nucleic Acids Research* **49**:10807–10817.
